# On the long-run efficacy of punishments and recommendations in a laboratory public goods game

**DOI:** 10.1038/s41598-017-12490-5

**Published:** 2017-09-25

**Authors:** Ananish Chaudhuri, Tirnud Paichayontvijit

**Affiliations:** 1 Department of Economics, University of Auckland, Auckland, New Zealand; 2 Department of International Political Economy and Development, Rangsit University, Bangkok, Thailand; 3 660 Owen G Glenn Building, 12 rafton Road, Auckland, 1142 New Zealand

## Abstract

We use decision-making experiments with human participants to study cooperation in a laboratory public goods game. Such games pose a conflict between cooperating, which is socially optimal and free-riding, which promotes individual self-interest. Prior research emphasizes the need for de-centralized peer-to-peer punishments as an evolutionarily stable response to the problem of free-riding, especially where interactions occur over long horizons. We show that a simple exhortative message appealing to participants’ goodwill can achieve high rates of cooperation in social dilemmas played over many rounds, even in the absence of punishments for free-riding.

## Introduction

Social dilemmas, such as voting, contributing to charities and public goods, managing common pool resources and even tackling global warming and climate change, pose a conflict between cooperating, which is socially optimal and free-riding, which promotes individual self-interest. There is a now a voluminous literature that uses decision-making experiments with human participants to study this issue^1,2^. A significant insight coming out of this work is that enforcing cooperation in social dilemmas requires punishments for free-riding. Such may be implemented by a central authority via a formal sanctioning system^3,4^. But, even in the absence or unavailability of formal sanctioning institutions, such punishments are often implemented informally via decentralized sanctions where norm violators are punished by their peers; even when such punishments are costly to inflict and interactions are short-lived, thereby conferring no future benefits to current punishments^5–7^. Indeed, evidence suggests that human subjects exhibit a preference for such informal peer-to-peer punishments over a more formal sanctioning system, especially where the latter is costly to implement and such informal punishments can be more efficient than formal mechanisms in sustaining cooperation over time^8,9^.

However, de-centralized peer-to-peer punishments have drawbacks. First, punishment becomes a second-level public good; those who are willing to punish, must not only punish free-riders but also those non-punishers, who may cooperate but do not punish free riders and hence free ride on others’ punishment and so on. This requires the creation of “meta-norms” of cooperation and punishment^10^. Second, while punishments can increase cooperation, their efficiency implications (net social benefit after subtracting punishment costs) are unclear and depends crucially on the cost-benefit ratio. It must be possible to inflict heavy punishment relatively cheaply, in order for punishments to be efficient^11,12^. Third, one often finds the incidence of “anti-social” punishments, where free-riders punish cooperators, often in anticipation of punishment from the latter. This can be seriously detrimental to efficiency^13,14^.

Gächter *et al*.^15^ show that, given a long enough horizon (where human participants play a linear public goods game with costly punishments for 50 rounds), punishments can be unambiguously beneficial. They go on to argue that such “altruistic” punishments over a long horizon are evolutionarily stable and provide support for group selection models of cooperation and punishment^16,17^. The very threat of punishment is often enough to sustain cooperation without much actual punishment being meted out, which explains the efficacy of punishments over the longer term^18^.

But, Gächter *et al*. compare their punishment treatment with a control treatment that does not allow for any punishment. They also look at “partners” matching where group composition remains unchanged over time. This begs the following questions: First, is mutual cooperation among humans impossible in the absence of costly punishments? There is evidence that exhortative messages, assortative matching and feelings of community are often successful in enhancing cooperation^2,19–22^. Might morally persuasive messages appealing to participants’ goodwill achieve a similar goal? Second, while fixed matching in small groups may be suitable for studying behavior in the ancestral past, how does that translate into our current, primarily urban, existence, where most interactions are with non-related strangers, which makes signaling intent or building reputations difficult? Punishment may be less effective, compared to other mechanisms such as public messages, when most of the interactions are short-lived.

While public service announcements may not be costless, it is likely that any such costs, monetary or societal, would be less than the cost of punishments, whether formal or informal. Antanas Mockus, ex-Mayor of Bogota, Colombia writes: “*…Bogotá’s traffic was chaotic and dangerous when I came to office…. Among various strategies, we printed and distributed hundreds of thousands of “citizens’ cards,” which had a thumbs-up image on one side to flash at courteous drivers, and a thumbs-down on the other to express disapproval. Within a decade, traffic fatalities fell by more than half*”^23^.

In Chaudhuri and Paichayontvijit (2011)^24^ (henceforth, referred to as Experiment 1) we present data from public goods experiments with paid human participants (n = 172) who interact in groups of four over 20 rounds. At the beginning of each round, each participant is given an endowment of 10 tokens (each token is worth NZ $0.05 for a total endowment of NZ $0.50) that they can either retain or contribute to a public account. Contributions to the public account are doubled and re-distributed equally among group members. Self-interest suggests contributing nothing to the public account, while the social optimum is for all participants to invest their entire token endowments into the public account in each and every round. This game is now standard in the behavioral economics tool-kit for studying the dichotomy between conflict and cooperation^1^.

Experiment 1 consists of three treatments – *recommendation*, *punishment* and *control*. In all three, participants first play the stage game described above for 10 rounds with no intervention. In the *recommendation* treatment, immediately prior to the 11^th^ round, the experimenter hands out a sheet of paper to each and every participant with an exhortative message asking participants to contribute their entire token endowment to the public account. In addition, the experimenter also reads this message out loud for everyone to hear prior to the start every round from Round 11 onward.

In the *punishment* treatment, in each round from Round 11 onward, after having seen the contributions of the other group members, participants can assign punishment points to them in whole numbers ranging from 0 to 10. Each point sent costs the sender $0.05 while it costs the receiver 10% of her earnings for the round. If a participant receives 10 points or more, she loses all her earnings for that round. Participants are not aware of the identity of punishers thereby ruling out targeted punishments as revenge, which typically results in lower efficiency^2,13,14^.

Behavior in these two experimental treatments is compared to a third, *control* treatment where participants stop at the end of the first 10 rounds and are then told to resume playing without any further instructions. Participants know from the beginning that there will be 20 rounds of play divided into two sets of 10 rounds as described above.

We implement two matching protocols – *fixed matching (partners) and random re-matching (strangers)*. In the latter case, in each round, groups of four are formed by randomly matching participants in a given session. These two types of matching protocols, constituting a “complete network”, are common in such experimental studies, where each participant can either punish or be punished by any other participant within the group. There is a large related literature that looks at “incomplete networks”, where participants are constrained to interact with one or more immediate neighbors but not all group members. The nature of network architecture and the ability to monitor and/or punish one or more members of the group has important implications for the ability of punishments to sustain cooperation over time as well as the efficacy of such punishments^25–30^.

In Experiment 1, we demonstrated that an exhortative message appealing to participants’ goodwill to contribute to the public good was successful in sustaining cooperation and did better than the punishment treatment, particularly when group composition is fixed over time. Both treatments did at least as well, if not better, in terms of efficiency, as the control treatment. We present more details below.

However, in this previous study participants play only for 10 rounds following an intervention. The question is: will the pattern of sustained cooperation in the recommendation treatment persist over a longer horizon? In response, we collect additional data by running Experiment 2 (n = 232), where participants interact for 30 rounds in two parts, the first part with 10 rounds and the second part with 20. Exactly as in Experiment 1, participants first play the linear public goods game for 10 rounds without any intervention. Any intervention is introduced prior to Round 11. We felt that running 20 rounds following an intervention should be adequate for stable patterns to emerge and be established.

In Experiment 2, we implement three treatments: *recommendation*, *punishment* and *recommendation with restart*. The *punishment* treatment is identical to that in Experiment 1, except that participants interact for 20 rounds in the second part rather than 10. The *recommendation* treatment is mostly similar to that in Experiment 1, except that the same exhortative message is read out prior to Rounds 11, 15, 19, 23 and 27 only, i.e., prior to every fourth round, as opposed to before every round in Experiment 1.

The third treatment is new and combines a *recommendation* with a *“restart”* (henceforth referred to as the *restart* treatment). It extends the *recommendation* treatment in the following way. Here, following the first 10 pre-intervention rounds, participants are told that they would play the game for 12 more rounds, with the *same message*, as in the *recommendation* treatment, read out loud every four rounds. Except, in this treatment, there is a *“surprise restart”* following the 12^th^ round. Once those 12 rounds are over, the participants are invited to stay back and play for another 8 rounds. The *same message*, as in the *recommendation* treatment, is read out loud, once before round 1 and once more before round 5 of the last set of 8 rounds. So, as with the *recommendation* treatment, participants in the *restart* treatment also play for 20 rounds following the intervention; except they are asked to play for 12 rounds to start with and then they are asked to play for another 8 rounds, which they did not know about before-hand. The exhortative message is read out loud before the start of Rounds 11, 15, 19, 23 and 27; except there is a surprise restart between Rounds 22 and 23. Prior studies have shown that such restarts can lead to renewed optimism and give rise to increased cooperation following the restart^31,32^. We do not run the control treatment in Experiment 2 given that Experiment 1 shows that both punishments and recommendation treatments do at least as well as the control treatment, if not better, in terms of efficiency.

As in Experiment 1, participants are fully informed about the total number of rounds. For the punishment and recommendation treatments, they know that they will play 30 rounds divided into two halves, the first one with 10 rounds and the second with 20 rounds. For the *restart* treatment, they know that they will play for 22 rounds; 10 rounds to start with and then another 12 with a break and that they may be asked to stay for a third part. Following the first 22 rounds, participants are asked to stay back for an additional 8 rounds. Participants are free to leave prior to this third part, but no one did. Once again, we implement two matching protocols – *fixed matching (partners) and random re-matching (strangers)*. Below, for the purposes of completeness and comparison we present results from both Experiments 1 and 2.

## Results

**Figure 1:**
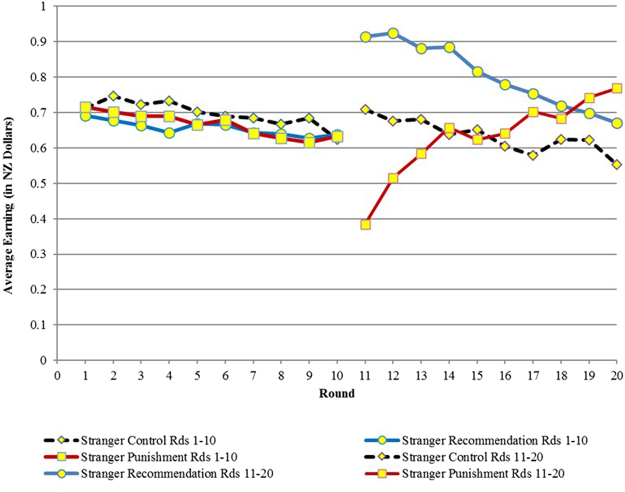
Earnings in Experiment 1 (20 round game) with strangers matching.

**Figure 2:**
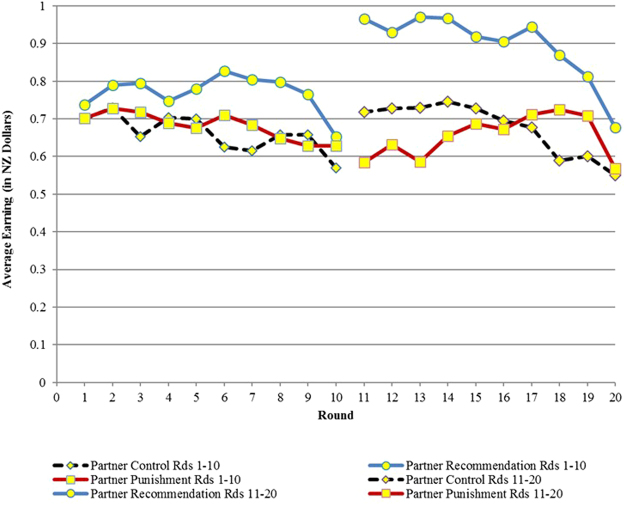
Earnings in Experiment 1 (20 round game) with partners matching.

**Figure 3:**
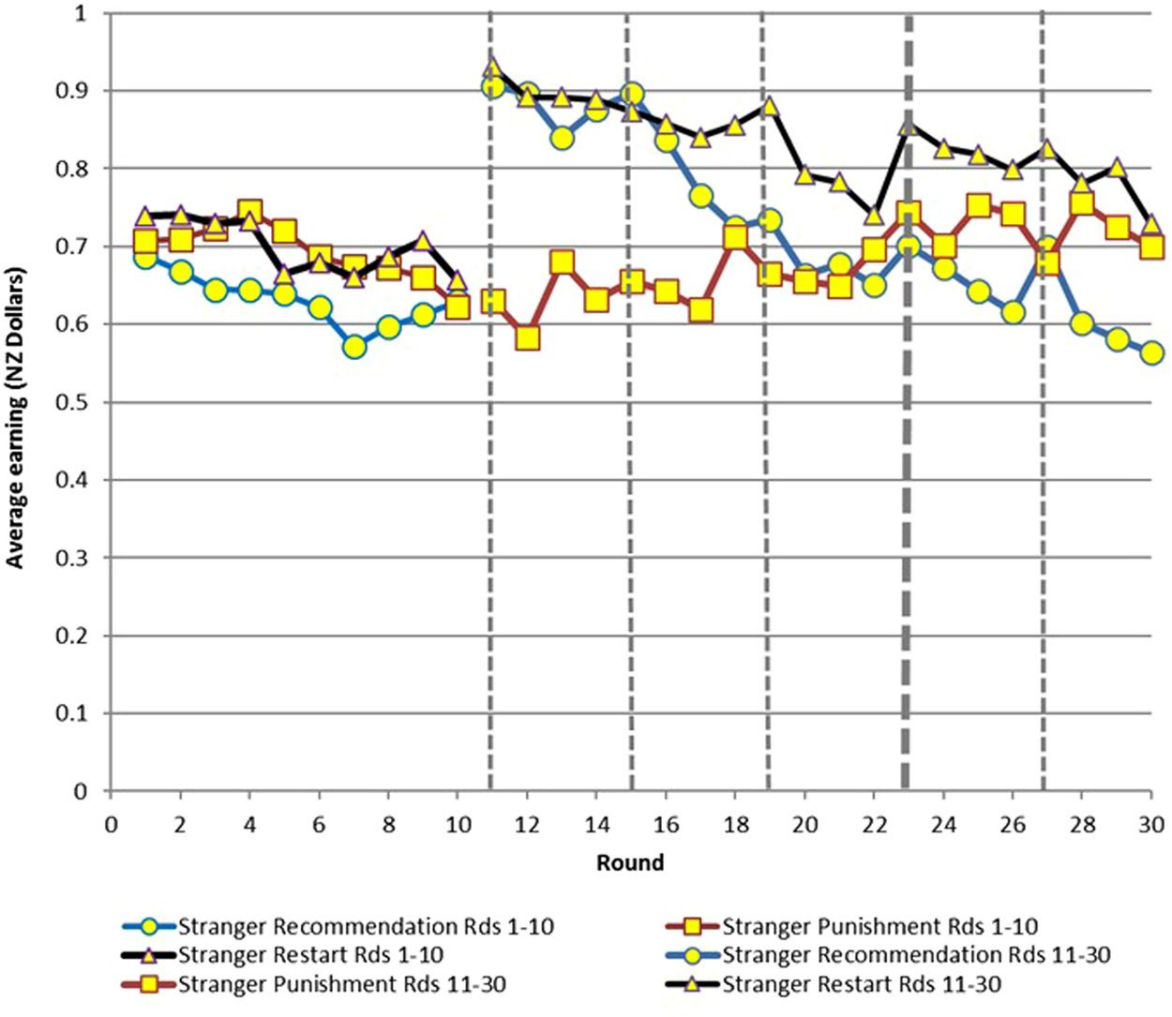
Earnings in Experiment 2 (30 round game) with strangers matching.

**Figure 4:**
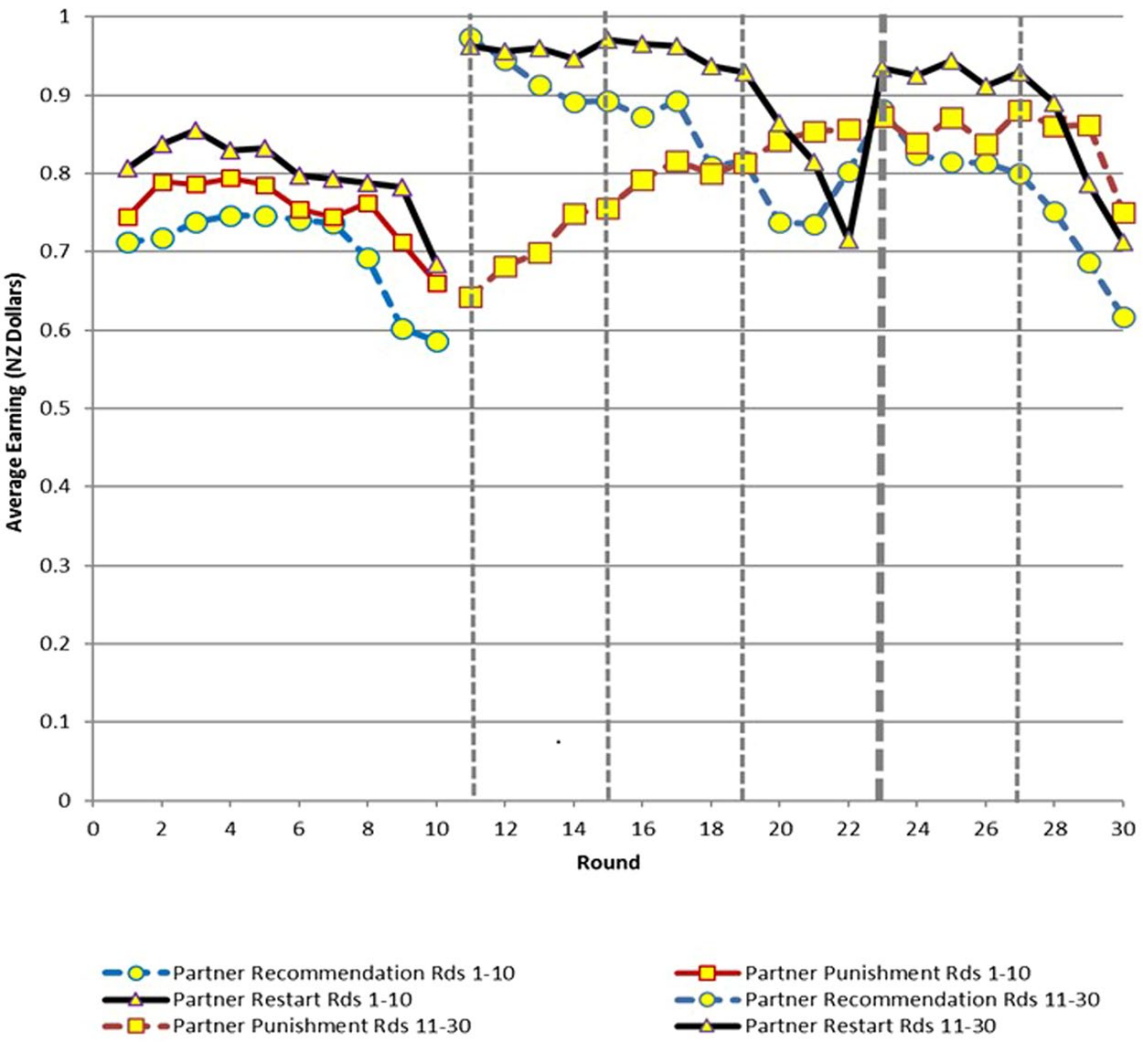
Earnings in Experiment 2 (30 round game) with partners matching.

Our main results in terms of efficiency are presented in Table 1 and Figs 1–4. Table 1 provides an overview of efficiency across treatments. Figures 1 and 2 show the results for Experiment 1, (reproduced from Chaudhuri and Paichayontvijit, 2011^23^) while Figs 3 and 4 do so for Experiment 2. *Efficiency is depicted on the vertical axis as earnings in NZ dollars*. In any round, if everyone contributes all 10 tokens to the public account, then each subject earns 20 tokens, which is equivalent to NZ $1. So the closer average earning per round is to NZ $1, the greater is efficiency. The efficiency numbers can also be interpreted as percentages: earnings of $0.80 imply 80% efficiency. In Figs 3 and 4, for Experiment 2, we use dashed vertical lines to depict the rounds with announcements. The thick dashed line indicates the *restart*, relevant only for that treatment.

**Table 1:** Overview of time trends in average earnings (efficiency).

Treatments	Stranger matching earnings (NZ $)			Partner matching earnings (NZ $)		
**Experiment 1 (20 round game):**						
	**Average**	**Round 11**	**Round 20**	**Average**	**Round 11**	**Round 20**
Control	0.63	0.7	0.55	0.68	0.72	0.55
Recommendation	0.80	0.90	0.67	0.90	0.95	0.68
Punishment	0.63	0.39	0.78	0.65	0.58	0.57
**Experiment 2 (30 round game):**						
	**Average**	**Round 11**	**Round 30**	**Average**	**Round 11**	**Round 30**
Recommendation	0.73	0.91	0.56	0.82	0.97	0.62
Punishment	0.68	0.63	0.7	0.80	0.64	0.75
Restart	0.83	0.92	0.72	0.90	0.91	0.7

Compared to punishments, recommendations have a more immediate effect on efficiency, particularly with partner matching. However, the influence of recommendations decays over time. Punishments, on the other hand, take time to get established but then generally exhibit an upward trend over time. Nevertheless, in Experiment 1, recommendations outperform punishments in terms of efficiency for at least 8 of 10 rounds if not more, with either partner or stranger matching. (Figs 1 and 2). Experiment 2 (Figs 3 and 4) provides more support for Gächter *et al*. (2008) in the sense that here punishments start to outperform recommendations later in the game. However, punishments still do not outperform the restart treatment, either with partner or stranger matching, even after 30 rounds.

In order to test whether differences in efficiency across treatments are significant, we rely on random effects regressions for individual earnings, with errors clustered on individual participants. Regression results are presented in Tables 4 and 5. (See Supplementary Materials.) For Experiment 1, the regressors include (1) round, (2) two dummies, one for the *recommendation* treatment and one for the *punishment* treatment with the *control* treatment as the reference category and (3) two additional dummies interacting the treatment dummies with round. The results suggest that compared to the *control* treatment, average contributions are higher in the *recommendation* treatment and *lower* in the punishment treatment, with either partner or stranger matching. For Experiment 2, where we no longer have a control treatment, the regressors include (1) round, (2) two dummies, one for the *recommendation* treatment and another for the *punishment* treatment, with the *restart* treatment as the reference category and (3) two additional dummies interacting the treatment dummies with round. Average earnings are lower in the *punishment* treatment, compared to either *recommendation* or *restart*, while there are no significant differences between the latter two treatments.

In all regressions, the coefficient for the punishment dummy is negative and significant, which implies that efficiency in this treatment starts out lower than the two comparison treatments (*control* and *recommendation* in Experiment 1; *recommendation* and *restart* in Experiment 2) but, over time, efficiency in the punishment treatment increases, compared to the other two treatments. This is shown by a positive and significant estimated coefficient for the interaction term between the punishment dummy and round and is true for both stranger and partner matching. These results are borne out by appropriate Wald tests comparing the time-trends. The implication is that while punishments take time to get embedded over time, depending on the parameter specifications, at some point in time, punishments will outdo recommendations. This is in line with Gächter *et al*. (2008) results. However, it is worth noting, that, on average, punishments do not outperform the other treatments, particularly the restart treatment, in terms of efficiency even after 30 rounds of interaction. (As a robustness check for these results, in Supplementary Materials we also provide results from one-way ANOVA with Bonferroni correction for multiple hypothesis testing).

## Discussion

These results suggest that messages appealing to human goodwill can be quite effective in enhancing cooperation compared to punishments, even with a moderately long horizon. However, the effectiveness of such messages vis-à-vis punishments may depend on the latter’s cost-benefit ratio, the length of the interaction horizon and the matching protocol. Punishments have greater impact when group composition is fixed than when interactions are short-lived. It is clear that there will always be some time horizon for which punishments will outdo other mechanisms in term of efficiency. But the efficiency enhancing properties of punishments take time to get established and appeals to human goodwill have a much more immediate impact and outdo punishments for extended periods. It is worth noting one caveat here: We did not implement a restart within the punishment institution as we did with a recommendation. Doing so may well have increased the efficacy of punishments. But, the question is not so much whether punishments are sufficient to deter anti-social behavior. Of course, they are. However, it is possible that, at times and across a range of social dilemmas, especially in the context of anonymous short-lived interactions, more benign mechanisms may achieve similar goals, at a lower social cost. We will leave the exploration of such approaches for further research.

## Materials and Methods

The study involves 404 participants recruited from the University of Auckland; mostly undergraduate business and economics students, who are inexperienced with the game. Experiments were conducted in the DECIDE laboratory at the university using the Veconlab website (http://veconlab.econ.virginia.edu) developed by Charles Holt at the University of Virginia. We report results from two separate experiments, Experiment 1, where participants interact for 20 rounds and Experiment 2, where they interact for 30 rounds. The data for Experiment 1 comes from our prior work in Chaudhuri and Paichayontvijit (2011)^24^. The experiments in this study were approved by the University of Auckland Human Participants Ethics Committee (UAHPEC) and all methods were performed in accordance with the relevant guidelines and regulations established by UAHPEC. All participants are adults and provided informed consent in writing prior to the start of a session. All participants are aware that they are free to withdraw at any point during the session without having to cite a reason.

In Experiment 1, participants are placed into groups of four and interact for 20 rounds. At the beginning of each round, each participant is given an endowment of 10 tokens. Each token is worth NZ $0.05. Each participant then decides how to allocate her token endowment either to a public account or a private account. All participants make their contribution decisions simultaneously and in whole tokens. Tokens contributed to the public account are doubled by the experimenter and redistributed equally among the 4 group members so that the MPCR is equal to 0.5. Participants get to observe the individual contributions made to the public account by their group members (without learning their identities) and their own earnings at the end of each round. This implies that if everyone makes full contribution to the public account then each subject will end up with 20 tokens or NZ $1.00 per round. (We use experimental cents in the instructions rather than saying that each token is worth NZ $0.05. This is because each token contributed to the public account will then be doubled in value to NZ $0.10. Redistributed equally among the four group members nets NZ $0.025 per player. However, the software does not allow three decimal points and rounds this up to NZ $0.03. As a result, we denote payoffs in experimental currency but then make the actual payoff equal to 50% of experimental payoffs).

There are three different treatments. In all three treatments the participants play the standard game as described above for the first 10 rounds with no intervention whatsoever. Any intervention is introduced prior to Round 11 and any further relevant instructions provided at that point. The first treatment is the one with a *Recommendation*. Here, immediately prior to the 11^th^ round the experimenter hands out a sheet of paper to each and every participant with the following message. In addition, the experimenter also reads this message out loud for everyone to hear prior to the beginning of every round from Round 11 onward.

*“You should contribute 10 tokens in each round. NOTICE* *that if all participants in a group follow the message then every participant will make 100% return on their contributions. For example, if in a particular round all 4 players in your group contribute all 10 tokens to the public account, then each group member will receive 20 tokens in return of their investment of 10 tokens. You will be helping yourself and everyone else in the group if you contribute all 10 tokens in every round.”*

The second treatment is the *Punishment* treatment. Here, from Round 11 onward, participants have the opportunity to punish group members. Each round consists of two stages. In the first stage, participants make their contribution decisions, in whole tokens. After everyone has made a decision, contribution decisions are revealed to all participants (by using identification numbers only).

In the second stage, participants can assign punishment points, in whole numbers, to group members. The cost of sending punishment points increases with more points sent, as shown in the third row of Table 2. For example, sending 3 punishment points costs 30 experimental cents (NZ $0.15). The recipient of punishment points has her earnings for the round reduced by the relevant fraction shown in the second row of Table 2. Each point received reduces payoff by 10%. So if a participant receives a total of 10 points or more, she will forfeit all her earnings for that round. Participants are provided with a sheet of paper containing this table. Participants do not get to learn the identity of those who may have punished them. This avoids the issue of targeted punishments and revenge seeking, which typically results in lower efficiency^2,13,14^.

**Table 2:** Costs to Sender and Receiver for each punishment point.

Punishment Points	0	1	2	3	4	5	6	7	8	9	10
Cost to Receiver (percent of payoff lost)	0	10%	20%	30%	40%	50%	60%	70%	80%	90%	100%
Cost to Sender (experimental $)	$0.00	$0.10	$0.20	$0.30	$0.40	$0.50	$0.60	$0.70	$0.80	$0.90	$1.00

The net payoff to participant *i* in each round of the punishment treatment is given by

$${\pi }_{i}=\,{\rm{\max }}[0,(10-{c}_{i}+0.5\sum _{j=1}^{4}{c}_{j}-sen{t}_{i})\times (1-\frac{receive{d}_{i}}{10})]$$

where 10 is the token endowment provided to each participant at the beginning of each round, *c*_*i*_ is contribution to the public account by participant *i*, 0.5 is the marginal per capita return for each token contributed to the public account, $$\sum _{j=1}^{4}{c}_{j}$$ is sum of contribution to the public account by all four players in the group, *sent*_*i*_ is the punishment points sent by participant *i*, and *received*_*i*_ is the total punishment points received by participant *i* in that round. This punishment technology is different from that employed by Gächter *et al*.^15^ where every $1 given up by the punisher results in a $3 loss in payoff for the punished. However, the choice of the cost-benefit ratio is essentially arbitrary and it is likely that there is interaction between the cost-benefit ratio and the time horizon; in the sense that some punishment mechanisms will outdo alternative mechanisms, in terms of efficiency, earlier rather than later.

Behavior in these two experimental treatments is compared to a third *Control* treatment where participants play all 20 rounds without any intervention. Here, participants are asked to stop at the conclusion of the 10^th^ round, at which point they are told that there are no further instructions and they should simply continue playing the same game.

At the end of the session a participant’s total earning in tokens is converted into cash at the rate of NZ $0.05 per token. The sessions lasted about one hour and on average participants earned NZ $17. In addition, participants were paid NZ $4 for showing up. The detailed instructions are shown in the Supplementary Materials.

In Experiment 2, participants interact for 30 rounds in two parts, the first part with 10 rounds and the second part with 20. As in Experiment 1, participants play the exact same linear public goods game for 10 rounds without any intervention. Any intervention is introduced prior to Round 11.

Here we implement three treatments: *punishment*, *recommendation* and *recommendation with restart* (henceforth referred to as *“restart”*). The *punishment* treatment is identical to Experiment 1, except that participants interact for 20 rounds in the second part rather than 10. The *recommendation* treatment is also similar, except that the public message is read out prior to Rounds 11, 15, 19, 23 and 27 only, i.e., once every four rounds, as opposed to before every round in Experiment 1. We read the message out loud once every four rounds rather than every round as in Experiment 1. We felt reading the message every round would be repetitive and tedious.

The third *recommendation with restart* (referred to as the “*restart*”) treatment is new. Here, following the first 10 pre-intervention rounds, participants are told that they would play the game for 12 more rounds, with the same message, as in the *recommendation* treatment, read out loud every four rounds. Except, in this treatment, there is a “surprise restart” following the 12th round. Once those 12 rounds are over, the participants are invited to stay back and play for another 8 rounds. The same message is read out loud before the 1^st^ and 5^th^ rounds in this third part. So, participants in the *restart* treatment also play for 20 rounds following the intervention; except they play for 12 rounds to start with and after that, they are asked to play for another 8 rounds, which they did not know about before-hand. The message is read out loud before the start of Rounds 11, 15, 19, 23 and 27; except there is a surprise restart between Rounds 22 and 23. Average earnings (excluding the show-up fee) are approximately $26.00.

Again, we implement two matching protocols: *partners*, where group composition is fixed for the entire time and *strangers*, where participants are randomly re-matched between rounds. We provide details of the experimental design for both experiments in Table 3 (Supplementary Materials).

Experiment 1 was conducted in 2010 while Experiment 2 was run in 2016. The undergraduate degree in New Zealand takes three years. So, the 6-year hiatus between the two studies minimizes any possibility of repeat participants or communication between participants in one study with those in another.

There is no deception. Participants are aware of number of rounds of play, matching method and payoff structure. Where there is a “surprise restart”, the instructions state that the experiment will consist of at least two parts: the first part with 10 rounds and the second part with 12 rounds. Participants are further told that following the conclusion of the second part, they may be asked to stay back for a third part and if so, then they will be provided with further instructions at that point. Please see the detailed instructions under Supplementary Materials.

## Electronic supplementary material

Supplementary Material
