# Supplementary material for: On the long-run efficacy of punishments and recommendations in a laboratory public goods game
Source: Sci Rep. 2017 Sep 25;7:12286. doi: 10.1038/s41598-017-12490-5 (PMC5612960; doi:10.1038/s41598-017-12490-5)
Supplement: Supplementary file 1 — Supplementary Material [file 41598_2017_12490_MOESM1_ESM.pdf]

Title: On the long-run efficacy of punishments and recommendations in a laboratory public goods game

Authors: Ananish Chaudhuri[1] and Tirnud Paichayontvijit[2]

Affiliations: [1] Department of Economics, University of Auckland, Auckland, New Zealand; 660 Owen G Glenn Building, 12 Grafton Road, Auckland 1142, New Zealand; a.chaudhuri@auckland.ac.nz. (*Corresponding author*)

[2] Department of International Political Economy and Development, Rangsit University, Bangkok, Thailand; tirnud.pai@gmail.com

Supplementary Material:

1. *Additional results*

Table 3 provides an overview of the experimental design and the details of all the different treatments for both **Experiments 1 and 2**.

In **Tables 4 and 5,** we present results from random effects regressions for individual earnings with errors clustered on individual participants. We provide a discussion of the regression results in the **Main Text**.

Tables 6 and 7 present results from one-way ANOVA with Bonferroni correction for multiple hypothesis testing. This is done primarily as a robustness check since ANOVA is a less conservative test for significance for the following reason. When groups are fixed, we can treat each group as an independent observation. But, when participants are randomly re-matched from one round to the next, this is no longer an option and in those cases, the session as a whole constitutes one independent observation. But if we treated a session as an independent observation, then we would be left with between 2 and 3 independent observations for each treatment. This is clearly far from ideal. Therefore, within each treatment, we treat the earnings of each individual participant as an independent observation for our analysis. This implies that for each treatment, we have as many observations as the number of participants in that treatment.

The results reported in **Tables 6** and **7** suggest the following. In **Experiment 1,** efficiency is significantly higher under recommendations than with punishments. (stranger matching averages: \$0.80 for recommendations; \$0.63 for punishments; $p < 0.01$; partner matching averages: \$0.90 for recommendations; \$0.65 for punishments; $p < 0.01$.). In fact, here punishments do not outperform the control treatment, either with stranger matching (average efficiency of \$0.63 for both control and punishment treatments; $p = 1.00$) or with partner matching (control average = \$0.68; punishment average = \$0.65; $p = 1.00$).

The results are similar in **Experiment 2**. Punishments do not outperform other treatments in terms of efficiency. (*stranger matching*: \$0.68 for punishment, \$0.73 for recommendation and \$0.83 for restart; *partner matching*: \$0.80 for punishment, \$0.82 for recommendation and \$0.90 for restart.) With *stranger matching*, efficiency under punishments (\$0.68) is not significantly different from that under recommendation (\$0.73), while it is significantly less than that under the restart (\$0.83) (diff = 0.11; $p < 0.01$). For *partner matching*, differences in efficiency between punishment (\$0.80) and recommendation (\$0.82) are not significant, while efficiency in the restart treatment (0.90) is significantly higher than that under punishment (\$0.80) (diff = 0.10; $p < 0.01$).

Table 3: Overview of Treatments

Matching Protocol	Rounds	Treatments	Rules of the game	Number of Participants	Number of groups
Experiment #1					
STRANGER	20	Control	20 rounds with no intervention	32	8
STRANGER	20	Recommendation	First 10 rounds with no intervention; recommendation for each of the last 10 rounds	32	8
STRANGER	20	Punishment	First 10 rounds with no intervention; punishment for each of the last 10 rounds	28	7
PARTNER	20	Control	20 rounds with no intervention	20	5
PARTNER	20	Recommendation	First 10 rounds with no intervention; recommendation for each of the last 10 rounds	20	5
PARTNER	20	Punishment	First 10 rounds with no intervention; punishment for each of the last 10 rounds	40	10
			Total	172	43
Experiment #2					
STRANGER	30	Recommendation	First 10 rounds no intervention; recommendation prior to Rounds 11, 15, 19, 23, 27	32	8
STRANGER	30	Punishment	First 10 rounds no intervention; punishment for each of the last 20 rounds	48	12
STRANGER	30	Restart	First 10 rounds no intervention; recommendation prior to Rounds 11, 15, 19, 23, 27; a restart prior to round 23	32	8
PARTNER	30	Recommendation	First 10 rounds no intervention; recommendation prior to Rounds 11, 15, 19, 23, 27	36	9
PARTNER	30	Punishment	First 10 rounds no intervention; punishment for each of the last 20 rounds	52	13
PARTNER	30	Restart	First 10 rounds no intervention; recommendation prior to Rounds 11, 15, 19, 23, 27; a restart prior to round 23	32	8
			Total	232	58

Table 4: Random effects regression for earnings in Experiment 1 (20 round game)

**Dependent variable: Earnings**	**Stranger Matching**	**Partner Matching**
	**Estimated coefficients**	**Estimated coefficients**
Recommendation dummy	0.52***<br>(0.06)	0.48***<br>(0.10)
Punishment dummy	-0.54***<br>(0.08)	-0.35***<br>(0.12)
Round	-0.03***<br>(0.01)	-0.04***<br>(0.01)
Recommendation dummy*Round	-0.03***<br>(0.01)	-0.01<br>(0.01)
Punishment dummy*Round	0.10***<br>(0.01)	0.06***<br>(0.01)
Constant	1.42***<br>(0.05)	1.57***<br>(0.09)
Wald χ^2	310.86	190.40
Number of observations	920	800
Number of participants	92	80
Wald test for equality of coefficients		
Punishment = Recommendation	χ^2 = 205.66<br>Prob > χ^2 = 0.00	χ^2 = 99.87<br>Prob > χ^2 = 0.00
Recommendation dummy*Round = Punishment dummy*Round	χ^2 = 79.29<br>Prob > χ^2 = 0.00	χ^2 = 22.48<br>Prob > χ^2 = 0.00

Note: ***,**,* denote significance at 1%, 5% and 10% levels respectively.
Standard errors are presented in parentheses.

Table 5: Random effects regression for earnings in Experiment 2 (30 round game)

**Dependent variable: Earnings**	**Stranger Matching**	**Partner Matching**
	**Estimated coefficients**	**Estimated coefficients**
Recommendation dummy	0.01<br>(0.08)	-0.07<br>(0.06)
Punishment dummy	-0.59***<br>(0.08)	-0.55***<br>(0.08)
Round	-0.01***<br>(0.005)	-0.02***<br>(0.004)
Recommendation dummy*Round	-0.02***<br>(0.006)	-0.01*<br>(0.005)
Punishment dummy*Round	0.03***<br>(0.006)	0.03***<br>(0.006)
Constant	1.82***<br>(0.07)	1.97***<br>(0.04)
Wald χ^2	182.33	117.91
Number of observations	2240	2400
Number of participants	112	120
Wald test for equality of coefficients		
Punishment = Recommendation	$\chi^2 = 97.94$<br>Prob > $\chi^2 = 0.00$	$\chi^2 = 33.48$<br>Prob > $\chi^2 = 0.00$
Recommendation dummy*Round = Punishment dummy*Round	$\chi^2 = 130.64$<br>Prob > $\chi^2 = 0.00$	$\chi^2 = 61.87$<br>Prob > $\chi^2 = 0.00$

Note: ***,**,* denote significance at 1%, 5% and 10% levels respectively.
Standard errors are presented in parentheses.

Table 6: One-way ANOVA with Bonferroni correction testing for differences in mean earnings (efficiency) across treatments in <u>Experiment 1</u> (20 round game)

	Stranger Matching Recommendation (Mean= \$0.80) (n = 32)	Stranger Matching Punishment (Mean = \$0.63) (n = 28)	Partner Matching Control (Mean = \$0.68) (n = 20)	Partner Matching Recommendation (Mean = \$0.90) (n = 20)	Partner Matching Punishment (Mean = \$0.65) (n = 40)
Stranger Matching Control (Mean = \$0.63) (n = 32)	**Diff =0.17 p < 0.01**	Diff = **-** 0.003 p = 1.00	Diff = 0.04 p = 1.00	**Diff = 0.26 p < 0.01**	Diff = 0.19 p = 1.00
Stranger Matching Recommendation (Mean = \$0.80) (n = 32)	---	**Diff = -0.17 p < 0.01**	**Diff = -0.13 p < 0.01**	Diff = 0.92 p = 0.09	**Diff = -0.15 p < 0.01**
Stranger Matching Punishment (Mean = \$0.63) (n = 28)	---	---	Diff = 0.05 p = 1.00	**Diff = 0.27 p < 0.01**	Diff = 0.23 p = 1.00
Partner Matching Control (Mean = \$0.68) (n = 20)	---	---	---	**Diff = 0.22 p < 0.01**	Diff = -0.23 p = 1.00
Partner Matching Recommendation (Mean = \$0.90) (n = 20)	---	---	---	---	**Diff = -0.24 p < 0.01**

Note: “diff” shows the difference between the mean of the row and column variables (row mean – column mean). Significance at 1% is shown in **bold** while significance at 5% is shown in *italics*.

Table 7: One-way ANOVA with Bonferroni correction testing for differences in mean earnings (efficiency) across treatments in Experiment 2 (30 round game)

	Stranger Matching Punishment (Mean = $0.68) (n = 48)	Stranger Matching Restart (Mean = $0.83) (n = 32)	Partner Matching Recommendation (Mean = $0.82) (n = 36)	Partner Matching Punishment (Mean = $0.80) (n = 52)	Partner Matching restart (Mean = $0.90) (n = 32)
Stranger Matching Recommendation (Mean = $0.73) (n = 32)	Diff =-0.5 p = 1.00	**Diff =0.11 p < 0.01**	*Diff =0.10 p = 0.03*	Diff =0.08 p = 0.11	**Diff =0.17 p < 0.01**
Stranger Matching Punishment (Mean = $0.68) (n = 48)	---	**Diff =0.15 p < 0.01**	**Diff =0.14 p < 0.01**	**Diff =0.12 p < 0.01**	**Diff =0.22 p < 0.01**
Stranger Matching Restart (Mean = $0.83) (n = 32)	---	**---**	Diff =0.01 p = 1.00	Diff =0.03 p = 1.00	Diff =0.07 p = 0.47
Partner Matching Recommendation (Mean = $0.82) (n = 36)	---	**---**	**---**	Diff =0.02 p = 1.00	Diff =0.08 p = 0.17
Partner Matching Punishment (Mean = $0.80) (n = 52)	---	**---**	**---**	**---**	**Diff =0.10 p < 0.01**

Note: "diff" shows the difference of the Mean of the row and column variables (row mean – column mean). Significance at 1% is shown in **bold** while significance at 5% is shown in *italics*.

2. Instructions

Instructions for Part 1

This is an experiment in economic decision-making. The University of Auckland has provided the funds to conduct this research. The instructions are simple. If you follow them closely and make appropriate decisions, you may make an appreciable amount of money. This money will be paid to you in cash at the end of the experiment. This money is in addition to the $4 show-up fee that you get. Please do not talk to each other during the experiment.

[20 round game]:

This experiment will consist of two parts. Each of these two parts will consist of 10 rounds. These two parts may or may not be similar in that the second set of 10 rounds may or may not be similar to the first set of 10 rounds. We will now tell you how the first set of 10 rounds will work. At the end of the first set of 10 rounds we will tell you either that the second set of 10 rounds will work the same way as the first set of 10 rounds or we will tell you that the second set of 10 rounds will work differently from the first set of 10 rounds. If we tell you that the second set of 10 rounds will be different from the first set of 10 rounds, then at that point we will also give you some further instructions about how the second set of 10 rounds will work.

[30 round game]

This experiment will consist of two parts. The first part will consist of 10 rounds and the second part will consist of 20 rounds. These two parts may or may not be similar in that the second set of 20 rounds may or may not be similar to the first set of 10 rounds. We will now tell you how the first set of 10 rounds will work. At the end of the first set of 10 rounds we will tell you either that the second set of 20 rounds will work the same way as the first set of 10 rounds or we will tell you that the second set of 20 rounds will work differently from the first set of 10 rounds. If we tell you that the second set of 20 rounds will be different from the first set of 10 rounds, then at that point we will also give you some further instructions about how the second set of 20 rounds will work.

[30 round game with restart]

This experiment will consist of at least two parts. The first part will consist of 10 rounds and the second part will consist of 12 rounds. These two parts may or may not be similar in that the second set of 12 rounds may or may not be similar to the first set of 10 rounds. We will now tell you how the first set of 10 rounds will work. At the end of the first set of 10 rounds we will tell you either that the second set of 12 rounds will work the same way as the first set of 10 rounds or we will tell you that the second set of 12 rounds will work differently from the first set of 10 rounds. If we tell you that the second set of 12 rounds will be different from the first set of 10 rounds, then at that point we will also give you some further instructions about how the second set of 12 rounds will work. Following the conclusion of the second part, we may ask you to stay back for a third part. If we do so, then we will provide you with further instructions at that point.

In the partner matching protocol, the next part reads as follows:

You will be in a group with 3 other participants in each round, i.e. you will be part of a 4-person group. You will not know the identity of the other three group members in any round. The composition of the group that you are in will remain unchanged for the entire experiment. This means that you will be playing with the same three players for the entire set of 10 rounds during the first part of the experiment.

In the stranger matching protocol, the next part reads as follows:

You will be in a group with 3 other participants in each round, i.e. you will be part of a 4-person group. You will not know the identity of the other three group members in any round. The composition of the group will change from one round to the next. Prior to the beginning of each round, you will be randomly re-matched with three other participants to form a new group. This makes it unlikely that you will be playing with the same three group members for more than one consecutive round.

At the end of the experiment your earnings from this game will be converted to cash at the rate of 1 experimental dollar = NZ $0.50.

Here is how the first part of the experiment, i.e., the first set of 10 rounds will work. In each round each of you will have 10 tokens. In each round, you can invest these tokens in a private account or the public account. You must make your token choices in whole numbers. Tokens put in the private account are worth 10 experimental cents each. But each token invested in the public account will create a return of 5 experimental cents for you and 5 experimental cents for each of the three other group members. One way to think about this is: each token that you invest will be **doubled** in value (i.e. from 10 experimental cents to 20 experimental cents) by the experimenter and then divided equally among all four group members giving each group member 5 experimental cents for that round. Each round starts with an endowment of 10 tokens and proceeds in the same way. Remember, each experimental dollar is equal to NZ $0.50 or 10 experimental cents is equal to NZ $0.05.

Once you log in to the computer you will be assigned a subject ID. Please make a note of this and write down this number on the top of each page of your instructions. You will be able to read this instruction again once you log in to the website.

We will pay you your earnings from the experiment at the end of the session. You are free to go once you have been paid. Your earnings are private information and we encourage you to keep this information private. If at any point you have any questions or problems, please raise your hand for assistance.

Instructions for the Punishment Treatment

In the second part of this experiment, you are allowed to punish other group members.

Costs to Sender and Receiver for each punishment point

**Punishment Points**	0	1	2	3	4	5	6	7	8	9	10
**Cost to Receiver (percent of payoff lost)**	0	10%	20%	30%	40%	50%	60%	70%	80%	90%	100%
**Cost to Sender (experimental $)**	$0.00	$0.10	$0.20	$0.30	$0.40	$0.50	$0.60	$0.70	$0.80	$0.90	$1.00

- **Punishment Points:** In each round, after investment decisions for all members of your group are revealed at the end of the round, you may send up to 10 **punishment points**, which may be directed at one or more members in your group. If you send no points, the cost to you is $0, and as you send more points, your cost increases, as shown in the bottom row of the table. For example, if you send 3 points to other members, this will cost you 30 cents. The others can also send points to you, and their sending costs are determined in the same manner. The number of points you can send depends on how much money you have available in your private account at that point. *Remember, each experimental dollar is equal to NZ 0.50 or 10 experimental cents is equal to NZ $0.05.*

- **Effect of Points Received:** Whoever receives points has her earnings for the round reduced by the relevant fraction shown in the second row of the table. If you send 1 point to another person, her earnings will be reduced by a factor **0.1**, which is equivalent to multiplying their earnings by **0.9**. For example, if the person that you sent 1 point to has earning for that round of 17 cents, then her earnings will reduce to 15.3 cents (17 x 0.9 = 15.3). If that person receives 1 point from you and 1 point from another person, then her earnings reduction factor is given in the column for 2 points: **0.2**, which is equivalent to multiplying her earnings by **0.8,** etc. When you receive points, your earnings are reduced in the same manner.

- **Earnings Adjustment:** If you send points, the cost of the total number of points that you send will be deducted from your earnings. If you receive points, your earnings (after costs have been deducted for points you sent) will be reduced by a factor that depends on the total number of points received.

Instruction for Recommendation and Restart treatments

[20 round game]

There are no new instructions for this second part of the experiment except that before you get started and at the end of every round we will provide you with the following announcement:

"You should contribute 10 tokens in each round.

NOTICE that if all participants in a group follow the message then every participant will make 100% return on their contributions.

For example, if in a particular round all 4 players in your group contribute all 10 tokens to the public account, then each group member will receive 20 tokens in return of their investment of 10 tokens.

You will be helping yourself and everyone else in the group if you contribute all 10 tokens in every round."

(A sheet with this message is passed out at the beginning of Round 11.)

[30 round game]

There are no new instructions for this second part of the experiment except that before you get started and at the end of every fourth round we will provide you with the following announcement:

---Same announcement as above---

(A sheet with this message is passed out at the beginning of Round 11.)

[30 round restart instructions]

There are no new instructions for this second part of the experiment except that before you get started and at the end of every fourth round we will provide you with the following announcement:

---Same announcement as above---

(A sheet with this message is passed out at the beginning of Round 11.)

[Following the conclusion of Round 12 in Part 2 of the restart treatment]

For today's session, we would like you to stay back and play the same game for another 8 rounds. The instructions are exactly the same as before. Before we start, we will read out loud the announcement again and read it one more time after the fourth round.

Are there any questions?

LOGIN INSTRUCTIONS

- Login to the computer (using your university user name and password).
- Open Internet Explorer (or any other web browser).
- In the browser's search window enter the following web address and press enter: http://veconlab.econ.virginia.edu/login.htm
- Your screen should say: 'Veconlab Participant Login Screen'
- Click on 'Login'.
- Your screen should say: 'Veconlab: Enter Session Name'
- **Enter the Session Name: The session name will be of the form "aicXX" where X stands for a number. The experimenter will tell you the exact session name.**
- Click on 'Submit'.
- Your screen should say: 'Veconlab Participant Login. Fill in the relevant boxes with your name and a password of your own choosing. Click on 'Continue'.
- The computer will assign you a Subject ID Number. Please write down your ID number and **the password** that you chose on the top of EACH page of your instructions in the space provided. It is important that you remember the password! This password will help us to go back and re-insert you into the session in case you accidentally close the window during the session.
- Please follow the instructions displayed on screen.
